# Acupuncture in the emergency department for pain management: A BraveNet multi-center feasibility study

**DOI:** 10.1097/MD.0000000000028961

**Published:** 2022-03-04

**Authors:** Jeffery A. Dusek, Gene A. Kallenberg, Robert M. Hughes, Alan B. Storrow, Christopher J. Coyne, David R. Vago, Arya Nielsen, Alison Karasz, Ryung S. Kim, Jessica Surdam, Tracy Segall, M. Diane McKee

**Affiliations:** a UH Connor Whole Health, University Hospitals, Cleveland, OH; b Department of Family Medicine and Community Health, Case Western Reserve University School of Medicine, Cleveland, OH; c Department of Family Medicine, University of California San Diego, La Jolla, CA; d Department of Emergency Medicine, Case Western Reserve University School of Medicine, Cleveland OH; e Clinical Decision Unit, University Hospitals, Cleveland Medical Center, Cleveland, OH; f Department of Emergency Medicine, Vanderbilt University Medical Center, Nashville, TN; g Department of Emergency Medicine and Department of Radiation Medicine and Applied Sciences University of California San Diego La Jolla, CA; h Department of Physical Medicine and Rehabilitation and Department of Psychiatry, Vanderbilt University Medical Center, Nashville, TN; i Department of Family Medicine and Community Health Icahn School of Medicine at Mount Sinai, New York, NY; j Department of Family Medicine and Social Medicine, Albert Einstein College of Medicine/Montefiore, New York, NY; k Department of Epidemiology and Population Health and Institute of Clinical and Translational Research, Albert Einstein College of Medicine/Montefiore, New York, NY; l Department of Family Medicine and Community Health, University of Massachusetts School of Medicine, Worcester, MA.

**Keywords:** acupuncture, emergency department, nonpharmacologic, opioid, pain

## Abstract

**Purpose:**

Pain accounts for up to 78% of emergency department (ED) patient visits and opioids remain a primary method of treatment despite risks of addiction and adverse effects. While prior acupuncture studies are promising as an alternative opioid-sparing approach to pain reduction, successful conduct of a multi-center pilot study is needed to prepare for a future definitive randomized control trial (RCT).

**Methods:**

Acupuncture in the Emergency Department for Pain Management (ACUITY) is funded by the National Center for Complementary and Integrative Health. The objectives are to: conduct a multi-center feasibility RCT, examine feasibility of data collection, develop/deploy a manualized acupuncture intervention and assess feasibility/implementation (barrier/facilitators) in 3 EDs affiliated with the BraveNet Practice Based Research Network.

Adults presenting to a recruiting ED with acute non-emergent pain (e.g., musculoskeletal, back, pelvic, noncardiac chest, abdominal, flank or head) of ≥4 on a 0-10-point Numeric Rating Scale will be eligible. ED participants (n = 165) will be equally randomized to Acupuncture or Usual Care.

At pre-, post-, and discharge time-points, patients will self-assess pain and anxiety using the Numeric Rating Scale. Pain, anxiety, post-ED opioid use and adverse events will be assessed at 1 and 4 weeks. Opioid utilization in the ED and discharge prescriptions will be extracted from patients’ electronic medical records.

Acupuncture recipients will asked to participate in a brief qualitative interview about 3 weeks after their discharge. ED providers and staff will also be interviewed about their general perspectives/experiences related to acupuncture in the ED and implementation of acupuncture in ACUITY.

**Results:**

Recruitment began on 5/3/21. As of 12/7/21: 84 patients have enrolled, the responsive acupuncture intervention has been developed and deployed, and 26 qualitative interviews have been conducted.

**Conclusion:**

Successful conduct of ACUITY will provide the necessary framework for conducting a future, multi-center, definitive RCT of acupuncture in the ED.

**Clinical Trials.gov:**

NCT04880733 https://clinicaltrials.gov/ct2/show/NCT04880733

## 1 Introduction

The manner in which pain is treated in the US remains a public health problem. Pain accounts for up to 78% of emergency department (ED) visits,^[1,2]^ where acute pain continues to be under or improperly managed.^[1,3]^ In 2012, U.S. healthcare providers prescribed 50 times more opioids than the rest of the world combined,^[4]^ reflecting a persistent national epidemic causing 130 deaths per day in 2018^[5]^ and a 30% increase in deaths from 2019 to 2020.^[6]^ By the end of 2020, opioid deaths increased by an additional 35%.^[7]^ Patients experience burdensome adverse effects while taking opioids, both major (respiratory distress) and minor (constipation, nausea/vomiting, dizziness, sedation, pruritus, and urinary retention).^[8]^ In a large study, 17% of opioid-naive patients prescribed opioids in the ED for acute pain were still receiving opioids 1 year later.^[9]^ Non-pharmacologic options that demonstrate feasibility, efficacy, and effectiveness are needed to treat pain and mitigate reliance on opioids.

The Joint Commission has urged caution regarding opioid use in hospitals,^[10]^ requiring accredited facilities provide nonpharmacologic therapy options for pain, with acupuncture being one option.^[11]^ Acupuncture has a low risk of adverse events. The National Institutes of Health (NIH) Consensus Statement on Acupuncture published in 1998 found that “the incidence of adverse effects is substantially lower than that of many drugs or other accepted procedures for the same conditions”.^[12]^ Systematic reviews and surveys suggest acupuncture is safe when performed by appropriately trained practitioners^[13–20]^ with infrequent minor side effects such as feeling relaxed, elated, tired or having sensation or itching at point of insertion.^[17]^ Rare and serious complications such as infection or pneumothorax are directly related to insufficient training.^[18,19,21]^

Acupuncture has been found to be superior to placebo/sham controls and usual care in the treatment of chronic pain (low back, neck, shoulder, osteoarthritis of the knee and headache/migraine), where 85% of benefit persisted at 1 year following care.^[22]^ It is a primary treatment option recommended for chronic low back and neck pain without serious pathology by the Global Spine Care Initiative.^[23]^ For acute/subacute low back pain, a common presentation in the ED, a systematic review^[24]^ supported the American College of Physicians recommendation of acupuncture as a first-line treatment.^[25]^ A recent Randomized Controlled Trial (RCT) conducted in Tunisia found acupuncture to be superior to parenteral morphine for acute pain relief in the ED with fewer adverse effects.^[26]^ Additionally, in a recent multi-center trial conducted in Australia, acupuncture was found comparable to pharmacotherapy for acute pain relief in select ED patients (migraine, ankle sprain and low back pain).^[27]^ When the details are reported in studies, acupuncture sessions for acute pain in the ED averaged 10 to 30 minutes^[28,29]^ with mean times of 23 to 24 minutes^[30,31]^ and did not disrupt ED course of care.^[30–32]^ Studies also report high acceptability for acupuncture by patients with acute pain in the ED when measured.^[31–33]^ While evidence from systematic reviews^[34–38]^ is encouraging for acupuncture in the ED for reducing acute pain and anxiety, most prior studies have been single-site RCTs and have produced heterogeneous results unlikely to lead to consensus.^[39]^ For these reasons, future, multi-center RCTs are warranted. In addition, prior studies did not include formal implementation strategies to understand potential challenges to incorporating acupuncture in the busy ED environment.

ACUITY is a multi-site, feasibility RCT that will develop a responsive, manualized acupuncture intervention, refine data collection procedures, implement a pilot RCT, and assess outcomes for barriers and challenges to implementation of acupuncture in the ED. Successful completion of this feasibility study will provide our team with the necessary materials and knowledge to conduct a future, multi-site, pragmatic, definitive RCT of acupuncture versus Usual Care for acute pain relief in the ED.

## 2 Method/design

### 2.1 Study design and overview

ACUITY will include 3 EDs associated with research sites (UH Cleveland Medical Center ED and Ahuja Medical Center (site 1), the Vanderbilt University Medical Center (site 2), and University of California-San Diego Hillcrest (site 3). The Data Coordinating Center (DCC) will be located at the Albert Einstein College of Medicine. The 3 recruiting sites and the DCC are members of the BraveNet Practice Based Research Network (PBRN),^[40,41]^ which includes 17 well-known US integrative medicine clinics in the US.

ACUITY will include 3 components. In the first component, we will develop manualized acupuncture intervention for use in the pilot RCT. In a second component, we will conduct a pilot RCT to refine the collection procedures and ensure that the study protocol can be uniformly deployed across 3 sites. In a third component, we will evaluate feasibility using enrollment records, structured observations of the intervention, and qualitative interviews with ED providers and staff to assess barriers, challenges and facilitators to implementation. Structured observation and qualitative interviews, with acupuncture patients and ED healthcare providers and staff, will be used to assess feasibility of implementing a study such as this within the ED setting and to assess participants’ perspectives and experiences during the recruiting phase in the EDs.

### 2.2 Interventions and duration

Acupuncture for pain management in the ED will be compared to Usual Care. The interventions will be administered during each participant's ED visit. During that time, pre-, post- and discharge assessments will be administered.

### 2.3 Participants and population

The target population for the RCT will be adults presenting to the ED with acute non-emergent (musculoskeletal, back, pelvic, non-cardiac chest, abdominal, flank or head) pain ≥4 on a 0-10-point Numeric Rating Scale (NRS) due to non-penetrating injury. A total of 165 study patients will be recruited study-wide (50 per site plus 5 pilot participants per site).

Additionally, approximately 30 (10/site) ED stakeholders will be interviewed about their perspectives and experiences related to implementation of the acupuncture study in the ED. These will include providers (physicians, nurse practitioners, physician assistants, and registered nurses) and ED staff employed for at least 1 year including the study period, as well as acupuncturists who are employed or credentialed by one of the participating institutions to perform acupuncture in the ED as part of the study, and study staff trained in consenting and recruiting.

### 2.4 Sample size

We justify the sample size of the ACUITY by first considering the sample size that would be required for a future UG3/UH3 multi-center, non-inferiority RCT. Per the National Center for Complementary and Integrative Health (NCCIH) guidance^[42]^ and the literature,^[43–45]^ we recognize that results from pilot/feasibility studies are not suitable for determining power for definitive trials. Rather, clinically significant differences are required for the power calculation.^[43–45]^ The verbally administered NRS has been validated and a minimum clinically significant change for acute pain intensity has been shown range from 1.3 (95% CI 1.0, 1.5)^[46,47]^ to 2.0.

For noninferiority trials, it is more conservative to use the lower estimate of clinical significance, which is 1.3. Following convention, we divide the minimally reported clinically significant change by 2^[48]^ to obtain a non-inferiority margin of 0.65 (=1.3/2). That is, to test whether Acupuncture group is at most 0.65 units inferior to the Usual Care group, a sample size of 510 (255 Acupuncture vs 255 Usual Care) will achieve 80% power to detect noninferiority. We will use a one-sided Mann–Whitney test with type 1 error 0.025.

Our power calculation assumes pain improvement between the Acupuncture and Usual Care groups is equivalent and that the actual score distributions are normal. We assumed a standard deviation of 2.52 for the Acupuncture group based on our previous collection of pain scores related to our previous single-site pilot acupuncture in the ED (unpublished), and 3.2 for the Usual Care group, an estimate drawn from a study measuring effects of intravenous morphine at 60 minutes in the ED.^[28]^ Using the same retention rate for the posttreatment Patient Reported Outcomes data collection of the previous single-site pilot study (∼78%), we anticipate the target sample increases to a total sample size of 654 (327 Acupuncture vs 327 Usual Care) for a future fully powered RCT. However, the sample size for any future study will need to be adjusted based on the rates of retention rate that we find in this ACUITY feasibility RCT.

Since the goal of ACUITY is to test feasibility of the study procedures previously used in the single-site pilot RCT, we plan to enroll 150 participants (50 per site). However, an additional 5 pilot subjects per site will be consented and brought through the study protocol but will not be counted toward the 150-subject enrollment. This enrollment number is both pragmatic for achieving our goals related to assessing feasibility, acceptability and fidelity, and is approximately 25% of the eventual sample size required for a future definitive non-inferiority study as noted above. Our ability to recruit about 25% of the anticipated sample size required for a future, definitive RCT would demonstrate our collective capabilities to recruit across 3 US EDs.

### 2.5 Screening and eligibility

**Figure 1:**
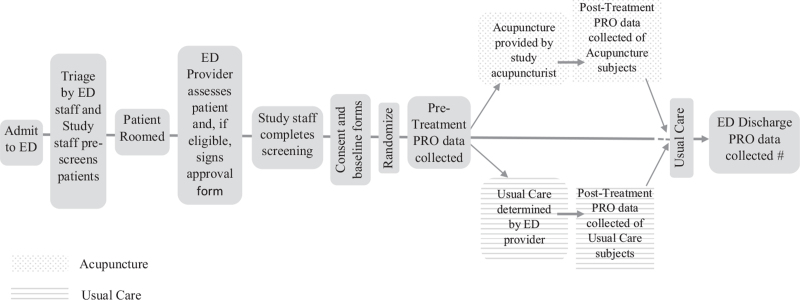
Flow of recruitment and data collection of study participants in the Emergency Department. #Pain Intensity and Anxiety PRO will only be assessed at ED discharge, if the time is >15 minutes from the Post-treatment time-point. PRO = Patient Reported Outcomes.

We will request a partial Health Insurance Portability and Accountability Act (HIPAA) waiver for prescreening. Therefore, patients will only be consented once screening is conducted and eligibility is confirmed. (See Fig. 1). Eligible participants for the RCT will be: 18 years or older, able to communicate in English, a level 3, 4, or 5 on the triage rating scale, have a chief complaint of acute musculoskeletal, back, pelvic, noncardiac chest, abdominal, and headache pain (≥4 on the NRS) due to nonpenetrating injury. Exclusion criteria for the RCT will be: fever exceeding 100°F, presenting with a chief complaint of a psychological/psychiatric concern, presenting with a chief complaint of migraine, current pregnancy, self-reported or documented opioid medication taken orally within 4 hours, presenting with chief complaint of joint dislocation, presenting with chief complaint of bone fracture, or confirmed or suspected COVID-19 infection.

Because pain levels and self-reported opioid use may not be documented on the triage notes, study staff will be able to approach patients who meet all other inclusion and exclusion criteria, after receiving the ED provider sign-off. After entering the patient's room and introducing the study, the study staff will collect the missing eligibility criteria and proceed accordingly.

Inclusion criteria for the qualitative interviews will be: ED providers (physicians, nurse practitioners, physician assistants, and registered nurses) and ED staff employed in the UH Cleveland Medical Center ED or Ahuja Medical Center (site 1), the Vanderbilt University Medical Center (VUMC) ED (site 2) and the University of California San Diego (UCSD) Hillcrest ED (site 3) for at least 1 year including the study period. Unlike any other previous study, we will interview the study acupuncturists who are employed or credentialed by one of the participating institutions to perform acupuncture in the ED as part of the study as well as patients who received acupuncture as part of the RCT to ascertain their perspectives on receiving acupuncture in the emergency setting. We may interview the study staff who recruited patients into the study. There are no exclusion criteria for the qualitative interviews.

### 2.6 Study flow

Enrollment for the RCT at each site is anticipated to require 6 to 9 months, with the last sites enrollment phase ending about 12 months after the first site begins enrollment. At each ED, the study staff and acupuncturist will work as a team, staffing an average 2, 6 hour recruitment/intervention sessions per week, varying the times and days across sites as this alteration may influence adoption.

As see in the Figure 1, when a patient arrives in the participating ED they will be triaged per current ED procedures. Within the triage unit, potential subjects will be identified/flagged by nursing staff and/or a member of the research staff. Patients who rate pain 4 or greater on the 0-10 NRS scale will be considered. At that time, the study staff will verify the patient's initial eligibility (age, understanding of English language, presence of pain) using the electronic health record (EHR). If the patient is potentially eligible, the study staff will approach the patient while waiting to be seen by their provider to hand them a patient recruitment flyer, if able.

The study staff will continue to monitor the patient's status and location within the ED using the EHR. The patient will then be roomed and seen by the ED clinical provider to ensure they do not need emergent care and have sufficient mental capacity to participate in the study. If the ED provider determines the patient does not have a clinical condition prohibiting study participation, the provider will sign a study approval form and give back to the study staff. Thereafter, the study staff will approach the potential subject to introduce the study.

### 2.7 Consent

Consent and study enrollment will happen shortly after screening given the nature of the ED environment and emergent nature of the patients’ acute pain. If the patient is interested in study participation after they have been assigned to a room, the study staff will assess capacity to consent. If the patient is capable then study staff will seek to obtain informed consent. Study staff will explain the study, including benefits, risks and interventions, and will stress to the patient that participation is voluntary and they may withdraw from the study at any time. During the structured field observation, at least 2, 6-hour shifts will be observed at each site during active patient recruitment and treatment. During consenting, the patient will be informed of the structured observation and given the opportunity to decline being part of this aspect of the study. Refusal will not affect the patient's continued care in the ED. The research staff will be trained to answer all questions that may arise. Only the participating patients themselves will be allowed to provide consent for inclusion into the study. No legally authorized representative may enroll a patient into the study.

If the patient is not interested in participating or refuses to provide their phone number and email for follow up assessments, a reason for refusal will be documented. Patients who decline participation will not be allowed to opt into the study for the remainder of their ED visit. However, they would be eligible to participate at a future ED visit if interested and if the study is still in the recruitment phase. If a patient enrolls in the study, he or she will no longer be eligible for study participation during future ED visits within the recruitment period. Remuneration for participation in the ED portion of ACUITY will be a $25 gift card and an additional $25 will be provided for those completing each of the 1 and 4 week assessments.

Participants who are assigned to the acupuncture group will be informed that they may be contacted to participate in a qualitative interview, which would be will recorded and transcribed. They may opt out of being contacted for the interview. Study staff from the DCC will contact the patients in the acupuncture group via email and/or phone within a week of their ED visit to schedule the interview and to share a study information sheet for them to review. All individuals who participate in the interview will receive an additional $25 gift card within 30 days of the completion of the interview.

Staff rosters will be used to identify physicians, nurse practitioners, physician assistants, nurses and staff who have worked in one of the EDs during the data collection period, to ensure familiarity with the study. Site credentialed study staff from the DCC or site specific study staff will contact these providers, whether in person during their site visit for the structured observation, or by email followed by phone, to invite providers to participate and to share the study information sheet for them to review.

### 2.8 Randomization

Random assignments will be made by the study statistician in permuted blocks of size 2 and 4. The block size will also be randomly generated to minimize correct prediction of assignments and preserve approximate balance between groups, using the rand function in R. Administrative personnel from the DCC will program a randomization tool in Research electronic data capture (REDCap) for study staff to use during the patient's baseline assessment.^[49]^

Assignment into the Acupuncture Arm or Usual Care Arm will be tracked by the study staff on the Screening and Enrollment log.

### 2.9 Intervention arms

Consistent with pragmatic trials,^[50]^ we have chosen to compare Acupuncture Therapy to Usual Care rather than to a sham acupuncture treatment. At this stage of investigation, we agree with the current consensus view in the acupuncture research community that research designs such as those used to study new pharmaceutical treatments are not adequate in the study of effectiveness for therapies like acupuncture.^[51]^ Further rationale for this choice stems from the consensus of the Department of Defense/NIH Acupuncture for the Treatment of Acute Pain Workshop.^[52]^ After two days of meeting with experts in statistics, acute pain and acupuncture, there was agreement from Department of Defense, Department of Veterans Affairs and civilian experts that design of acupuncture studies should compare acupuncture plus usual care to usual care alone. Our proposal is also consistent with the NIH Collaboratory recommendation for pragmatic trials.^[53]^

#### 2.9.1 Developing the acupuncture intervention

##### 2.9.1.1 Manualization

A major study goal is the development of an acupuncture intervention for use in ACUITY and in subsequent randomized controlled trials. The process of forming a consensus-based intervention protocol, sometimes called manualization^[54–56]^ describes one such adaptation that seeks to strike a balance between standardization and flexibility in acupuncture research.^[55]^ The Delphi process, developed by the RAND Corporation, is widely used for convergence of expert opinion within certain topic areas.^[57–59]^ We briefly describe the process below and a separate manuscript^[60]^ describes the development of the acupuncture intervention in detail. The Standards for Reporting Interventions in Controlled Trials (STRICTA) checklist was used as a guide.^[61]^

Briefly, we convened a group with expertise in acupuncture for acute pain to participate in a modified Delphi process on consensus steps and staging of an acupuncture intervention. This process adapts the Medical Research Council's guidance of 2000^[62]^ and 2008^[63]^ in developing and evaluating complex interventions with interacting components.

The manualized protocol will promote standardization as well as flexibility based on the acupuncturist's assessment of the patient's presentation within a predetermined framework and contextual considerations such as accessibility of various parts of a patient's body. Since there will be various pain presentations (musculoskeletal back, neck, limb pain; abdominal or flank pain; headache etc.), the acupuncture intervention will adhere to the manualized protocol.

##### 2.9.1.2 Acupuncture intervention and procedures

The acupuncture therapy intervention will be based on staging an interview/conversation, palpation, and selection of points or methods for treatment based on presenting factors. The acupuncturist will record the specific acupuncture points, the number of needles used, length of needle retention if applicable, length of session time, any limitations on session time, points used for extended auricular therapy, if applicable, and response from point stimulation based on parameters of the responsive manualized protocol. A consensus of common points utilized for each acute pain condition will be provided to the acupuncturist for reference during the acupuncture intervention. For each acute pain condition, the acupuncture experts also approved additional points that could be used at the discretion of the acupuncturist treating the patient. Hand rotation and perturbation of needles to de qi status will be allowed; electrical stimulation or moxibustion will not be included in this trial. Needle retention time can vary and may range from 5 to 40 minutes, but commonly will be 15 to 30 minutes. Total session time may vary due to the patients’ tolerance of the treatment, acupuncturists’ assessment of the patient or workflow consideration of the ED, such as a patient needing to leave the room for imaging to be performed.

At each ED site, acupuncture intervention will be provided by one of 2 licensed acupuncturists with the option of a third backup acupuncturist for flexibility in coverage. Acupuncturists will be nationally board certified and remain current with the National Commission for the Certification of Acupuncture and Oriental Medicine^[64]^ which includes passing of infection control standards exam; they will also have a valid and current state acupuncture license.

Each patient randomized to the Acupuncture arm will be provided no more than 1 treatment per admission to the ED. Due to workflow considerations of the ED of this pragmatic RCT, it is possible that some patients randomized to the Acupuncture arm of the study will not receive acupuncture. Acupuncturists will attempt to provide the acupuncture intervention as close to the beginning of the patient's ED visit as possible, after evaluation by the attending ED provider. The rationale for this decision is to help evaluate the use of acupuncture as a first line intervention for pain relief. Sterile, single-use non-coated acupuncture needles will be used for this study. Needles in sizes of .22 × 13 mm, .25 × 25 mm, .25 × 40 mm, and .30 × 40 mm, and will be available. The choice of needle size used will be left to the discretion of the acupuncturists.

Extended therapy pressure will be included via ear seeds, retained on auricular acupuncture points in order to extend the treatment benefit after ED care. We will use vaccaria seed with latex adhesive, and have a non-latex alternative for patients with latex allergy. Use and location of the ear seeds will be at the discretion of the acupuncturist. Patients will be asked to keep the seeds on their ears after discharge from the ED, and are directed to leave them on until they fall off or become uncomfortable. Ear seeds can be peeled off and thrown into the trash. Acupuncturists will provide the participant with the Acupuncture Post Care information sheet at the completion of the acupuncture treatment. This sheet has information about the ear seeds and general after care instructions.

As the study is taking place in the active clinical ED, the responsible ED clinician will have the ability to prescribe any medications or interventions to maintain the health of a participant regardless of the study treatment assignment. For those in the acupuncture group, as per the discretion of the responsible ED clinician pain medications will be delayed until after completion of acupuncture intervention.

##### 2.9.1.3 Early termination of intervention

A subject's participation at any time without their consent for the following reasons:

- If participation appears to be medically harmful to them;
- If subjects fail to follow directions for participating in the study;
- If it is discovered that the subject does not meet the study requirements;
- If the study is canceled; or
- For administrative reasons, including competitive enrollment (e.g., the target number of subjects has already entered the study.)
- If the patient's clinical condition worsens emergently as determined by physician

#### 2.9.2 Usual care

Patients assigned to the Usual Care arm will receive care and treatment for pain and any other symptoms or conditions as would usually be provided in the ED, in accordance with the relevant pain management and care policy at each participating ED. Furthermore, we have designed our study so that the medications provided to Usual Care patients will likely have enough time to take effect serving as a more pragmatic control to the Acupuncture arm than sham acupuncture.

#### 2.9.3 Concomitant interventions

##### 2.9.3.1 Allowed interventions

As the study is taking place in the active clinical ED, the responsible ED clinician will have the ability to prescribe any medications or interventions to maintain the health of a participant regardless of the treatment assignment of the participant.

##### 2.9.3.2 Required interventions

Acupuncture is required for those in the treatment group

##### 2.9.3.3 Prohibited interventions

For those in the treatment group, pain medications will be delayed until after completion of acupuncture intervention.

### 2.10 Data collection

#### 2.10.1 Clinical trial

ACUITY outcome assessments will include patient reported outcomes (PROs) such as self-reported pain intensity and anxiety on the 0-10 NRS (See Table 1). The use of self-reported pain ratings to assess pain is standard clinical practice.^[65,66]^ Although self-report of pain intensity is subjective, a reliable physiological measure to quantify pain has not been identified.^[67]^ These PROs will be collected via tablet computer in the PHI-approved data collection tool (REDCap). The study staff will hand the patient a tablet for confidential self-administration. Once the patient completes the questionnaires, the answers will be masked so the research staff will not have access to the scores. To keep research study staff blinded to scores, the data collection tool will be designed so that the study staff will only be able to determine that valid pain and anxiety scores were entered by the patient.

**Table 1:** Timetable of outcome measurements.

		Data collection time point			
Variables	Measure/Source	Pretreatment	Post-treatment	ED discharge	1-week and 4 follow-up
Assessments					
Pain Intensity	*Numeric Rating Scale, 0-10*	X	X	X#	X
Anxiety	*Numeric Rating Scale, 0-10*	X	X	X#	X
All pain medications in the ED and prescriptions	*EHR*			X	
Pain medication and/or opioid use	*Self-report*				X
Baseline variables of interest					
Demographics	*EHR data and self-report*	X			
Current Medications	*Self-report*	X			
Opioid use – last 30 days		X			
Previous acupuncture use		X			
ED visits in last year		X			
Other variables of interest					
Expectancy	*Self-report*	X			
Satisfaction questions			X	X	X
ED impact (ACUPUNCTURE PATIENTS ONLY)				X	

Outcome assessments by time-point. # Pain Intensity and Anxiety will only be assessed at ED discharge, if the time is >15 minutes from the Post-treatment time-point.

Demographic and baseline data will be collected both by the study staff and through EHR data extraction at baseline and will be entered directly into REDCap study database. All baseline and PostPROs (pain intensity and anxiety) will be entered directly by the study participant on a tablet via REDCap, such that all study staff (including PIs) are blinded to these scores. Satisfaction data will be collected as part of Post-treatment, ED-discharge and at 1-week and 4-week follow up for patients.

PostTreatment Pain Assessment will be collected within 60 minutes (+/- 15 minutes) of the Pre-Treatment score, thus including the acupuncture and usual care periods. Based on pharmacokinetics of the most common pain medications used in emergency care, 1 hour is the maximum time to effectiveness for any pain medication currently administered in most EDs.^[68]^

ED Discharge Assessment (ED Discharge) will be obtained within 15 minutes of patients’ discharge from the ED, for both acupuncture and usual care patients. If the study staff's shift has ended and it has been longer than 15 minutes since collecting posttreatment scores, study staff will collect discharge scores from the participant and leave for the day. However, discharge scores will not be attempted to be collected if the participant is discharged within 15 minutes of when post treatment scores were collected. This latter instance will not be considered missing data.

EHR data will be electronically extracted by each site with HIPAA protected identifiers as limited datasets. The data extracted from EHR will be verified by Medical record number and date with our electronic platforms. EHR extracted data will include demographic data, the ED triage note, any pain medication use in the ED, and opioid prescriptions at discharge.

One-week and 4-week follow-up Assessments (PROs, pain medication/opioid use, Adverse Event monitoring) will be collected either directly from the participant through the electronic data collection tool (via text message prompt) or collected on paper survey and entered by a member of the research team if the follow-up is completed over the phone. These various methods will be used to optimize participant retention. All 1-week (+/- 4 days) and 4-week (+/- 4 days) contacts will be conducted by a research staff who did not interact with the patient during their study participation in the ED.

Upon completion of study enrollment, members of the study team will verify the information contained within the database. After answering all queries in the database, the information contained will be locked and exported for analysis. Both the source (electronic data collection) data and exported databases will be stored as required by the respective rules and regulations (e.g., HIPAA authorizations will be maintained for at least 6 years).

### 2.11 Data collection implementation evaluationobjectives and statistical analysis plan

#### 2.11.1 Primary objective

The primary objective of the current protocol is to conduct a feasibility RCT to refine data collection procedures for a future, definitive RCT. We will evaluate the feasibility of research procedures including data quality completeness and participant recruitment and retention.

##### 2.11.1.1 Data completeness

Using study records and administrative data, we will track recruitment (proportion of eligible patients recruited), document recruitment rates (time to recruit intended sample), and rates of loss to follow up. Data collected at each time point will be evaluated for quality and completeness. In addition, all quantitative data analyses will be preceded by extensive data checking and verification to identify and resolve the reasons for missing values, inconsistencies, and out-of-range values. Although we anticipate some missing data based on our experience, we will carefully examine whether missingness is completely at random, at random or informative. Models proposed for analysis can handle incomplete data but do require at least that missingness be at random. Modelling will consider using multiple imputation techniques of covariates to reduce potential biases.

##### 2.11.1.2 Recruitment/Retention

To assess recruitment, we will track the number of eligible patients presenting to the ED during the time of enrolling sessions and the proportion who agree to participate. Basic demographics and presenting complaint will be collected for all eligible patients, allowing us to identify subgroups who are more or less likely to participate. Similarly approaches will be used to assess rates of loss to follow up at all data collection points. We will assess variables across different patient groups (including age, race, sex), and across the study arms as well as overall and by sites.

#### 2.11.2 Secondary objectives

The secondary objectives of this study are to: develop a responsive acupuncture intervention and assess implementation in EDs associated with 3 BraveNet research sites for acceptability and fidelity. This study will include qualitative interviews and structured observation to assess feasibility of implementation.

To evaluate implementation of the acupuncture intervention, using both quantitative data (study records, stakeholder surveys) and qualitative data (interviews and observations), we base our selection of implementation outcomes on the synthesis and recommendations outlined by Proctor et al in their authoritative 2011 paper.^[69]^ We include all of the early phase outcomes, excluding cost, proposed in this widely cited framework, including: Feasibility (“practicability”), provider adoption, perceptions of appropriateness, acceptability, and treatment fidelity. These will be assessed both quantitatively and qualitatively. In addition to implementation outcomes, we will also examine implementation processes, strategies, stakeholder experiences, and barriers and facilitators to implementation using both qualitative interviews and real-time observations.

##### 2.11.2.1 Structured observation

Observations will be focused largely on the influences of actors and contextual factors on implementation uptake/feasibility. Examples could include: disruptive behavior or manifestations of severe illness/pain; staff strategies to mitigate or address these in order to facilitate treatment uptake; provider statements or interactions that interfere with uptake; environmental variables (a “slow night” in the ED that results in extra rooms to be used for the intervention, or the difference between a weekend and weekday night); etc. Observation notes will form the basis of a complete detailed field observation to be analyzed along with interview data using the strategies described below.

##### 2.11.2.2 Qualitative interviews

Patients randomized to the acupuncture group will be contacted to participate in a brief qualitative interview about 3 weeks after their ED visit. We expect 15 patient participants per site (n = 45 total) will consent to the qualitative interviews about their experience of receiving acupuncture as part of the RCT.

A semi-structured interview guide will be created to focus on likely implementation barriers and facilitators, including interactions and communications with the research team, patient flow, burden of the intervention, perceived need and benefits of the intervention, etc. As described above, we will use the domains of the Consolidated Framework for Implementation Research (CFIR) to organize the topic guides that will be developed for the qualitative interviews.^[70]^ Interview recordings will be stored on a secure server and interviews will be professionally transcribed.

### 2.12 Quantitative analysis

#### 2.12.1 Feasibility of recruitment and retention

Recruitment rates (# enrolled / # eligible), pace of accrual, data completeness, and retention will be assessed at each data collection point, overall and across sites. We will assess variables across different patient groups (including age, race, sex), and across the study arms.

Provider adoption will be assessed by the proportion of their eligible patients that clinicians approve for study participation. We will assess variables across different provider characteristics (including age, race, sex), and across the study sites. We will also track completeness of data collection, and patterns and proportions of missing data at each time point. We will compare these proportions overall and across sites and by demographics as described above.

#### 2.12.2 Acceptability

To assess patient acceptability, at ED discharge, 1-week and 4-week follow-up, all participants will be asked to answer “How satisfied are you with how your pain was managed during your ED visit” and “Overall how satisfied are you with your treatment during your ED visit?” each on the 5-point Likert Scale.

To assess ED provider acceptability, after each sites’ enrollment period is complete, ED staff will be asked to complete a brief survey (via REDCap) to assess their general satisfaction with acupuncture as a treatment in the ED by answering “Do you view acupuncture in general as an appropriate intervention for the ED setting?” (Likert scale (1-very appropriate—5-Very inappropriate) and “Do you view acupuncture in general as helpful in managing patient pain in the ED?” (Likert scale (1-Very helpful—5- Not at all helpful). To assess ED providers’ general satisfaction with how the ACUITY research was delivered in the ED, we will ask providers “Were you satisfied with how the ACUITY acupuncture intervention was delivered in your setting?” (Likert scale (1-Very satisfied—5-Very dissatisfied) and “Did the ACUITY project impose a burden on ED staff in your setting?” (Likert Scale 1 – Not a burden, 2 – Somewhat of a burden, 3 – Moderate burden or 4 – Extreme burden).

There will be comparisons across sites and across patient groups (e.g., pain location, patient demographics, etc.). We will calculate the proportion of subjects answering each question at 95% exact CI across sites as well as across patient groups such as different presenting complaints and patient demographics. Variables will be assessed across different provider characteristics (including age, race, sex), and across the study sites.

#### 2.12.3 Fidelity

To assess fidelity, we will determine the proportion of patients who are treated in a manner consistent with the manualized intervention and treatment fidelity parameters determined by consensus of acupuncture experts. We expect treatment fidelity measures will include: the dose (minimum number of needles, minimum points treated, minimum duration of needle retention time), and the delivery (was the intervention delivered as planned or cut short due to ED flow).^[57]^ Acupuncture documentation will be reviewed of all acupuncture participants and to assess manual fidelity. Additionally, the review will evaluate the proportion of cases when acupuncturists treated the patient “off manual” and their reasoning, as well as assess whether minimum standards for number of needles used, points treated, and needle retention time were met. Assessment will be across different practitioners, times of day, sites, and by participant demographics (e.g., sex and pain complaints).

### 2.13 Qualitative analysis

To analyze interview and observational data, we will use NVivo (https://www.qsrinternational.com/nvivo-qualitative-data-analysis-software/home/), a computer program that facilitates

1. the rapid organization and retrieval of thematically linked data; and
2. the use of quantitative grouping variables to classify cases and generate complex comparisons (. In a first step of thematic analysis, a preliminary coding scheme will be developed and applied to a subset of the data and then revised as needed.

This process will be repeated on subsets of data until it is judged sufficiently accurate and comprehensive. Data will be uploaded into NVivo and coded. Memos will be created for each interview describing the major themes emerging in the transcript. Next, coded data will be retrieved and used to create case summaries of key themes related to implementation processes at each site. Quantitative implementation outcome data will be used where relevant to create groups for the purpose of comparison. Within-site analyses will include comparisons of relevant groups (e.g., comparing perceptions of the intervention across different genders and ethnic groups; patients who achieve satisfactory pain relief vs those who do not; providers who are more vs less satisfied with the protocol, among others). In the cross-site analyses, we will examine differences in barriers, facilitators and implementation processes across sites with the goal of generating inferences regarding the important factors shaping differences in implementation outcomes (e.g., presuming sites differ on rates of recruitment, examining our qualitative data to generate hypotheses regarding the causes of this documented difference).

Lessons learned from field observation and preliminary analysis of process implementation at each site may inform implementation at subsequent sites. The success of program implementation depends on many factors, as described in Damschroder et al's influential CFIR framework.^[70]^ The CFIR includes 5 “domains” influencing implementation: the intervention, inner and outer setting, individuals, and processes. We will use the CFIR domains to structure our inquiry and to make sure that our qualitative data collection instruments, coding system, and interim reports reflect the complex multiple levels of influence that will shape the conduct and outcomes of the acupuncture intervention.

### 2.14 Quality assurance

All study staff will complete Collaborative Institutional Training Initiative or NIH Human Subjects training prior to commencement of study activities. Quality Assurance activities will be conducted at each subject study visit, as well as on a monthly, quarterly and annual schedule, and on an as-needed basis in response to staff or process changes.

The study staff will review and complete the Eligibility Checklist for each enrolled participant. Before randomization, the study staff will review the consent documentation and confirm adherence to the consent processes. At visit completion, the study staff will complete the ED Visit Checklist that captures the required elements of the visit. Queries and alerts, generated by the electronic data capture system, occurring during the clinical visit will be corrected as soon as notified during data entry or as soon as time allows following the visit.

Quality assurance activities for Qualitative Interview visits will begin by verifying that the participant is appropriate for the qualitative interview process. For both patient and provider qualitative interviews, the interviewer will confirm the subject's eligibility and willingness to participate. At visit completion, the interviewer will complete the Interview Visit Checklist that captures the required elements of the visit.

On a monthly basis, the site principal investigator (PI) will review newly executed consents using the Quality Assurance Participant Data Review Tool and new Eligibility Checklists and source documentation. On a quarterly basis, the Site Coordinator (SC) or designee will work with the Multi-Site Coordinator (MSC) to review 100% of the site's executed consents and review completion and accuracy of the source documents and the eCRFs for 100% of subjects at the site. The SC/MSC will also review query reports to confirm manual and automatic queries have been resolved. Training Logs will be reviewed by the SC/MSC every 3 months to verify training is current and properly documented. This will include a review for institution-specific and protocol-specific trainings. The Site Regulatory Binders will be updated by the SC when changes to licenses, certifications, credentials, Institutional Review Board (IRB) documents or Curriculum vitaes are made during the study; the MSC will review these binders quarterly. This review will be documented and summarized in the ACUITY Essential Documents Review Tool. At least annually, the MSC will conduct a complete review of each Site Regulatory Binder.

Study staff will document any protocol deviation after becoming aware of the event. The site PI or their delegated research staff will review and sign off on the deviation and designate it as major or minor. The site PI or delegated staff is responsible for reporting the deviation according to the IRB of record's guidelines.

### 2.15 Ethics

The IRB at University Hospitals Cleveland Medical Center (UH-CMC) will serve as the single IRB for ACUITY. The protocol and all amendments have be approved by the UH-CMC IRB (STUDY20200618) and any changes will be transmitted to the recruiting sites and the DCC.

The study protocol and subsequent changes to the protocol will be uploaded to ClinicalTrials.gov. Individual Data Use Agreements will cover data sharing between the enrolling sites and the DCC. A separate Data Use Agreement will cover data sharing from the DCC to the Case Western Reserve University (CWRU)/UH site (Dr. Dusek and his team) for oversight and report preparation to the NCCIH.

### 2.16 Data storage

Any data, specimens, forms, reports, video recordings, and other records that leave the site will be identified only by a participant identification number (Participant ID, PID) to maintain confidentiality.

All electronic data will be kept in password protected databases behind an electronic firewall at sites and then securely sent to the DCC and maintained on password protected PCs by a designated data manager. As above, the DCC will securely share data collected at their site and data received from the other 2 sites with Dr. Dusek and team at CWRU/UH. Data shared with CWRU/UH will be stored in a password protected database behind an electronic firewall on a secure UH server. Data in hard copy form will be stored in locked file cabinets within a secure, badge-access location at each site. Upon completion of study enrollment, members of the study team will verify the information contained within the database. After answering all queries in the database, the information contained will be locked and exported for analysis. Both the source (electronic data collection) data and exported databases will be stored as required by the respective rules and regulations (e.g., HIPAA authorizations will be maintained for at least 6 years).

Information will not be released without written permission of the participant, except as necessary for monitoring by IRB, the US Food and Drug Administration, the NCCIH, and the Office for Human Research Protections.

### 2.17 Patient safety

In the event a patient experiences an adverse event (AE), they will be treated by providers in the site ED and will have access to emergency response equipment as necessary. Based on our successful prior experience with our observational study and RCT, the likelihood of adverse physical, psychological, social, and legal risks is expected to be very small. Acupuncture carries very slight risks for bleeding, bruising, fainting (acushock), and needling pain. Acupuncture involves inserting thin, sterile needles in the skin. The needles are not inserted into the skin very far. Sometimes the needles cause slight discomfort or minor bleeding. Any acupuncturist who provides treatments in this study will be licensed by the state in which they practice. Monitoring of the study for AEs will be continuous throughout the study. In the event of an AE or Serious Adverse Event, it will be reported to the UH IRB in compliance with UH IRB standards.

### 2.18 Data and safety monitoring

As the current study is not a definitive RCT, a formal data and safety monitoring committee is not required by NCCIH. However, an Independent Monitoring Committee (IMC) will consist of several experts in the field of Complementary and Integrative Health. Specifically, the IMC will include a physician/researcher, a biostatistician and an expert in study design and implementation of acupuncture research in clinical practice and an expert in acupuncture, clinical trials and biostatistics. The IMC members are not associated with ACUITY, are not part of the key personnel involved in this grant, and have not collaborated with the 2 PIs, within the past 3 years. The IMC members are qualified to review the patient safety and data generated by ACUITY and membership approved by the NCCIH.

### 2.19 Study discontinuation

The study may be discontinued at any time by the IRB, the NCCIH, the Office for Human Research Protections, the US Food and Drug Administration, or other government agencies as part of their duties to ensure that research participants are protected. Study doctors or study sponsor may stop a participant from continuing in the study without their consent if it appears to be harmful to the participant, if they fail to follow directions for participating, if it is determined that they do not meet study requirements, if the study is cancelled, for administrative reasons, or if the patient's clinical condition worsens.

## 3 Discussion

Well-designed pragmatic trials are needed to clarify the feasibility and effectiveness of acupuncture for acute pain in the ED and its impact on opioid utilization. Pragmatic clinical trials (PCTs) are done in real-world clinical settings with generalizable populations to generate actionable clinical evidence at a fraction of the typical cost/time needed to conduct a traditional clinical trial.^[50,71]^ PCTs are part of the NIH's vision for bridging the gap between research and care,^[50,53]^ and are supported by the Center for Medicare & Medicaid, Patient Centered Outcomes Research Institute, PBRNs and community-based participatory research initiatives across the Federal government.^[72]^ Designed to inform clinical decisions and improve practice and policy, PCTs engage patients, practitioners, and health system communities. Classical efficacy RCTs such as “traditional randomized controlled trials” compare interventions against a control using rigid study protocols and minimal variation in a highly defined and carefully selected population. In 17 years, only 14% of tRCT research findings led to widespread changes in care.^[71,73]^ The NIH Collaboratory on pragmatic trials recommends early/ongoing stakeholder engagement.^[50]^ Thus, our study uses a mixed methods implementation evaluation which will

1. carefully assess feasibility of research procedures and
2. evaluate implementation outcomes (feasibility, adoption, appropriateness, acceptability and treatment fidelity); and
3. collect interview and observational data with the goal of understanding barriers and facilitators to implementation.

Successful completion of the multi-site feasibility RCT will provide the necessary framework for conducting a future, multi-site RCT of acupuncture therapy compared with usual care in pain patients in EDs across the BraveNet PBRN using UG3/UH3 mechanism. Completion of the feasibility RCT (R01) and the subsequent completion of a future definitive RCT (UG3/UH3) will provide critical evidence to support inclusion of acupuncture as a readily available treatment in EDs across the United States. Such an expansion would provide Americans with additional non-pharmacologic methods for comprehensive pain care and ideally reduce patients’ opioid use.

Implementing an RCT in the ED during a pandemic will hold unique challenges. With hospital and ICU bed use at all-time highs nationwide, the impact of COVID-19 may limit resources and our accessibility to recruit study participants within EDs. Available space within ED's high turnover operational areas, for example super track, fast track, urgent care, etc., may not have the capacity to offer a dedicated space for acupuncture treatment. We foresee these higher turnover areas as being the primary source of participants in the study as patients with a lower acuity (3, 4, or 5) and included chief complaints will be triaged into these areas. Our manualized acupuncture model allows for acupuncturists to remain flexible within the flow of the patient's diagnostic and ED treatment plan, when choosing a location for patient acupuncture treatment, and with needle placement. Fostering a collaborative, working relationship with the EDs administrators will help keep communication channels open in the event recruiting needs to be suspended due to rising COVID-19 numbers, or if changes need to be made to accommodate rising ED utilization.

### 3.1 Study status

The ACUITY study has been registered on www.clinicaltrials.gov (May 11, 2021) and all study protocols have been approved by the IRB at UH, protocol version 5. Study staff at each site have been trained on recruiting and data collection procedures and administering the manualized acupuncture intervention.

As of December 7, 2021, the UH CMC ED and Ahuja Medical Center (Site 1) has recruited all 55 participants from their site. The recruitment process took 6 months and 2 weeks, or 72 sessions, to complete, with 432 patients screened. As of December 7, 2021, the UCSD Hillcrest ED (Site 3) has been recruiting for 2 months and 1 week, or 28 sessions, with 241 patients screened, enrolling 29. VUMC (Site 2) has as anticipated start date of January 11, 2022.

The patient qualitative interviews have been completed at Site 1 with 12 acupuncture participants contributing their perceptions for this portion of the study. Five provider qualitative interviews at Site 1 have been completed. The remaining have an anticipated completion date of January 2022. At Site 3, 2 acupuncture participant qualitative interviews are complete and 7 provider interviews are complete. Completion of the remaining qualitative interviews for Site 3 is in progress, with a completion date to coincide with the completion of their recruiting phase. Qualitative interviews for Site 2 will commence with their recruiting phase.

### 3.2 Dissemination plan

In accordance to NIH policy, the authors will ensure that results of ACUITY will be submitted to www.clinicaltrials.gov. Informed consent documents for the RCT will include a specific statement relating to posting a study summary and results of the RCT information on ClinicalTrials.gov.

The ACUITY team is committed to widespread dissemination of study results via presentations at national and international conferences. Publication of results will be governed by the policies and procedures developed by the ACUITY Executive and Steering Committees. Any presentation, abstract, or manuscript will be made available for review by the sponsor and the NCCIH prior to submission. Members of the DCC will have access to the final dataset. Through a Data Use Agreement between the DCC and CWRU/UH site, Dr. Dusek will also have access to the final dataset. When applicable,

ACUITY data will be uploaded to appropriate public repositories.

## Acknowledgments

The authors would like to thank the following individuals for their considerable contribution to ACUITY:

CWRU/UH Site: Megan Quesada LAc, Christine Kaiser DACM LAc

UCSD Site: Erin Raskin DACM LAc, Amanda Walker MA, Daisy Cruz BA, Alejandra Reyes LAc

VUMC Site: Karen Miller RN MPA, Alexandra Dimidik MS, Chongbin Zhu PhD

Data Coordinating Center: Claudia Lechuga MS, Qi Gao PhD, Afrida Khurshid BA.

## Author contributions

**Conceptualization:** Jeffery A Dusek, Gene A Kallenberg, Robert M Hughes, Alan B Storrow, Christopher J Coyne, David R Vago, Arya Nielsen, Alison Karasz, Ryung S Kim, M Diane McKee.

**Data curation:** Arya Nielsen, Alison Karasz, Jessica Surdam, Tracy Segall.

**Formal analysis:** Alison Karasz, Ryung S Kim.

**Funding acquisition:** Jeffery A Dusek, M Diane McKee.

**Investigation:** Jeffery A Dusek, Gene A Kallenberg, Robert M Hughes, Alan B Storrow, Christopher J Coyne, David R Vago, Arya Nielsen, Alison Karasz, Jessica Surdam, Tracy Segall, M Diane McKee.

**Methodology:** Jeffery A Dusek, Arya Nielsen, Alison Karasz, Ryung S Kim, M Diane McKee.

**Project administration:** Jeffery A Dusek, Jessica Surdam, M Diane McKee.

**Writing – original draft:** Jeffery A Dusek, Gene A Kallenberg, Robert M Hughes, Alan B Storrow, Christopher J Coyne, David R Vago, Arya Nielsen, Alison Karasz, Ryung S Kim, Jessica Surdam, Tracy Segall, M Diane McKee.

**Writing – review & editing:** Jeffery A Dusek, Gene A Kallenberg, Robert M Hughes, Alan B Storrow, Christopher J Coyne, David R Vago, Arya Nielsen, Alison Karasz, Ryung S Kim, Jessica Surdam, Tracy Segall, M Diane McKee.
